# Scaling of buccal mass growth and muscle activation determine the duration of feeding behaviours in the marine mollusc *Aplysia californica*

**DOI:** 10.1242/jeb.246551

**Published:** 2024-04-15

**Authors:** Stephen M. Rogers, Jeffrey P. Gill, Ana Skalski De Campos, Katherine X. Wang, Isha V. Kaza, Victoria X. Fan, Gregory P. Sutton, Hillel J. Chiel

**Affiliations:** ^1^ Department of Life Sciences, University of Lincoln, Brayford Pool Campus, Lincoln LN6 7TS, UK; ^2^ Department of Biology, Case Western Reserve University, Cleveland, OH 44106-7080, USA; ^3^ Departments of Biology, Neurosciences, and Biomedical Engineering, Case Western Reserve University, Cleveland, OH 44106-7080, USA

**Keywords:** Muscle force, Elastic force, Buccal mass, Allometry, Micro-CT, Biting, Swallowing, Slow movement

## Abstract

The mechanical forces experienced during movement and the time constants of muscle activation are important determinants of the durations of behaviours, which may both be affected by size-dependent scaling. The mechanics of slow movements in small animals are dominated by elastic forces and are thus quasistatic (i.e. always near mechanical equilibrium). Muscular forces producing movement and elastic forces resisting movement should scale identically (proportional to mass^2/3^), leaving the scaling of the time constant of muscle activation to play a critical role in determining behavioural duration. We tested this hypothesis by measuring the duration of feeding behaviours in the marine mollusc *Aplysia californica* whose body sizes spanned three orders of magnitude. The duration of muscle activation was determined by measuring the time it took for muscles to produce maximum force as *A. californica* attempted to feed on tethered inedible seaweed, which provided an *in vivo* approximation of an isometric contraction. The timing of muscle activation scaled with mass^0.3^. The total duration of biting behaviours scaled identically, with mass^0.3^, indicating a lack of additional mechanical effects. The duration of swallowing behaviour, however, exhibited a shallower scaling of mass^0.17^. We suggest that this was due to the allometric growth of the anterior retractor muscle during development, as measured by micro-computed tomography (micro-CT) scans of buccal masses. Consequently, larger *A. californica* did not need to activate their muscles as fully to produce equivalent forces. These results indicate that muscle activation may be an important determinant of the scaling of behavioural durations in quasistatic systems.

## Teaser

**Summary:** As *Aplysia* grows, biting behaviour duration is determined by the time constant of muscle activation rather than mechanical forces. Swallowing movements are faster because the jaw muscles grow disproportionally larger.

## INTRODUCTION

How does size affect the neural control and performance of behaviour? All animal movements are the sum of inertial, elastic, viscous and gravitational forces but the relative contribution of these different forces depends critically on both size and the rate of movement. The mechanics phase shift (MPS) framework (Sutton et al., 2023) combines size and behavioural cycle time to determine the dominant forces acting during behaviour and thus predicts how neuromuscular control should be organised, regardless of the phylogeny and anatomy of different species. The MPS has been successfully applied to legged locomotion in large, fast animals (horses – dominated by inertial forces) and small, slow animals [stick insects (Phasmatodea) – dominated by elastic forces; Sutton et al., 2023]. In principle, any cyclical behaviour can be interpreted in the same analytical framework, and doing so will identify the key mechanical forces upon which neuromuscular control must act. Sutton et al. (2023) did not explore how growth, which affects the size of moving structures, alters forces during behaviour. One aim of the current paper was to analyse how the scaling of mechanical forces affects behaviour in a specific system: feeding in the Californian sea hare *Aplysia californica*, a herbivorous marine mollusc, which grows from approximately 150 mg to over 1 kg (Audesirk, 1979).

The second objective was to determine how non-mechanical factors affect the scaling of behaviour. The MPS framework identifies the physical forces and mechanical constraints on behaviour, but behaviour is also shaped by physiological processes that show greater variation between species (Sutton, et al., 2023). For example, muscle contraction shows substantial scale-dependent variation, including in muscle shortening velocity (Hill, 1950; Medler, 2002; Marx et al., 2006), muscle deactivation rate (Marsh, 1990), inertial resistance due to muscle mass (Ross et al., 2020; Ross and Wakeling, 2021) and the time constants of muscle activation (James et al., 1998; Van Wassenbergh et al., 2007; Ross et al., 2018). In legged or winged locomotion across taxa, maximum muscle velocity scales negatively with mass (Medler, 2002), but no evidence was found for such a relationship in swimming or non-locomotory behaviours (Medler, 2002). Inertial scaling of muscle properties is unlikely to affect behaviours dominated by quasistatic forces. It is difficult to predict how muscle activation will change with animal and muscle size; this needs to be empirically measured.

The MPS framework makes a clear prediction that slow movements in smaller animals should be dominated by elastic (quasistatic) forces. The stability to perturbation of quasistatic systems implies that neural control need not change with animal size (McMahon, 1975; Hooper; 2012; Clemente and Dick, 2023). *Aplysia californica* is an ideal animal in which to test the effects of scaling in an elastic force-dominated system because it is slow moving and shows little change in bodily proportions, despite undergoing considerable growth, allowing behaviours to be measured throughout its lifespan. *Aplysia* has already served as a model system for the analysis of learning and memory (Susswein et al., 1986; Baxter and Byrne, 2006; Hawkins et al., 2006), motivated behaviour (Hurwitz and Susswein, 1992; Morton and Chiel, 1993a,b) and motor control (Cropper et al., 2017; Church and Lloyd, 1994; Webster-Wood et al., 2020; Gill and Chiel, 2020) because of the tractability of its nervous system to detailed analysis. After metamorphosing from a free-swimming veliger and assuming their slug-like form, *A. californica* remain nearly morphologically identical as they grow through several orders of magnitude (Kriegstein et al., 1974), engaging in similar feeding behaviour throughout their lives.

The kinematics, biomechanics and neural control of the mouthparts, or buccal mass, has been intensively studied in *Aplysia* (Drushel et al., 1997, 1998; Neustadter et al., 2002; Sutton et al., 2004a,b; Novakovic et al., 2006; Webster-Wood et al., 2020). The buccal mass is a muscular tube-like structure containing a ball-like grasper (odontophore), which bears a toothed radula (Howells, 1942). During feeding, the grasper protracts and retracts, while the radula alternately grips and releases seaweed. Differences in the phasing and extent of these characteristically slow movements produce different behaviours: biting is exploratory, with strong protraction and weak retraction of the grasper, aiming to contact and draw new food items into the buccal mass; during swallowing, food is ingested by being moved through the buccal mass to the oesophagus and consists of strong retraction movements alternating with moderate protraction (Kupfermann, 1974).

Analysing the scaling of behaviour requires that the scaling of mechanical forces be separated from the scaling of physiological processes. To understand the scaling of feeding behaviour in *Aplysia*, we therefore addressed the following questions.

First, what are dominant mechanical forces during feeding in *Aplysia* throughout its lifetime? We estimated the energy allocated to inertial, viscous and elastic forces by considering the mass, mean velocity and displacement of the grasper, tissue stiffness and viscosity of feeding movements in large and small *A. californica*. Second, how does growth affect the time it takes for muscles to activate and reach their maximum force during feeding? We answered this question by allowing *A. californica* to attempt to feed on inedible seaweed strips, producing an *in vivo* approximation of isometric muscle contraction. Third, we used micro-computed tomography (micro-CT) scans of small and large *A. californica* buccal masses to determine whether the relative proportions of large and small buccal masses are fundamentally the same. Could deviations from predicted behavioural changes with scale be explained by allometric growth of the buccal mass? Addressing these questions provides new insights into the neural control of feeding in *Aplysia*, and more generally how size affects the control and performance of behaviours.

## MATERIALS AND METHODS

### Animals

*Aplysia californica* Cooper 1863 ranging from 8 to 1424 g were obtained from Marinus Scientific (Long Beach, CA, USA) and housed in a 189 l tank equipped with filters, aerators and artificial seawater (ASW, Instant Ocean, Cincinnati, OH, USA) at a constant 16±1°C with a 12 h/12 h light/dark cycle. To promote vigorous feeding behaviours, animals were starved immediately upon receipt from the supplier for a period proportional to their mass prior to experiments: small animals (<100 g) were starved for 3–5 days, large animals (≥100 g) were starved for 7–9 days.

### Temperature control

**Fig. 1.**
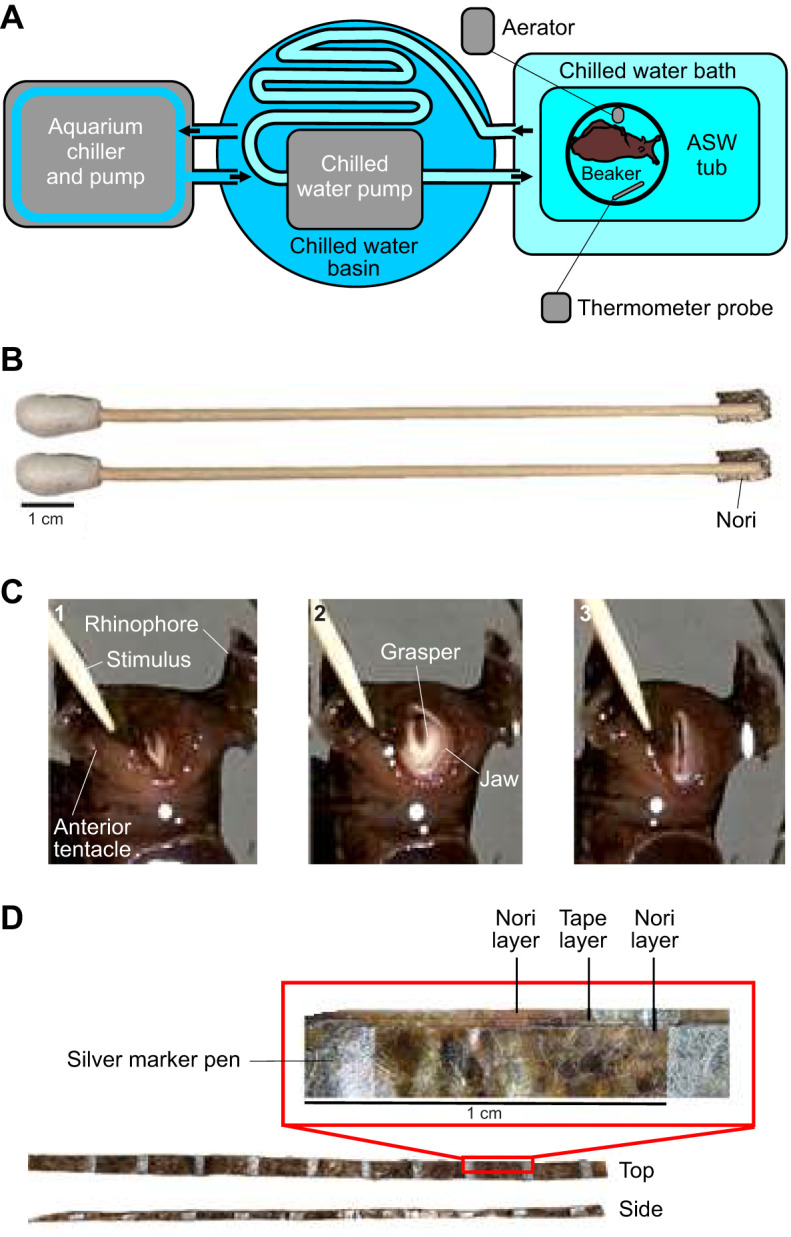
**Cooling system and feeding stimuli.** (A) Schematic diagram of the two-stage cooling system used to maintain a stable experimental temperature of 16±0.1°C. ASW, artificial sea water. (B) Biting stimuli consisted of a piece of nori held in a cleft in a wooden dowel placed against the rhinophore (olfactory organ), anterior tentacle and peri-oral zone. (C) Three video stills showing the progress of a bite in *Aplysia californica*: (1) opening of the jaws; (2) full protraction with grasper clearly visible; (3) end of retraction. (D) Unbreakable seaweed strips (top and side views; inset: layers) were made by placing nori on both sides of double-sided adhesive tape marked at 1 cm intervals with a silver marker pen.

Feeding responses are sensitive to temperature. Pilot data showed that even a 1°C increase in temperature caused measurable decreases in overall bite and swallow durations (data not shown). Therefore, an active cooling system was used to maintain temperature within the range of 16.0±0.1°C (Fig. 1A). A pump circulated tap water between an aquarium chiller (TECO Model CA 200, Ravenna, Italy) and a chilled water basin at 12.5°C. A second pump circulated another, isolated, tap water supply through hoses immersed in the chilled water basin to a second large plastic (∼10 l) bath: the hoses allowed for heat exchange between the isolated water supply and the chilled water basin. This second bath was, in turn, used to cool a ∼5 l container filled with artificial sea water (ASW). Another beaker or small bucket, proportional to the size of an experimental animal, was immersed in the ASW container to confine the animal's movement. Finally, an aerator and a thermometer probe were placed near the animal in the inner container to improve the oxygen supply, to promote water mixing and to measure temperature.

### Feeding stimuli

Feeding stimuli proportional to the animals' size were prepared in advance. Three types of stimuli were used: biting, unloaded swallows and loaded swallows.

Biting stimuli, designed to provide appropriate chemosensory stimuli to elicit biting movements of the grasper, consisted of small pieces of dried nori (Deluxe Sushi-Nori, nagai roasted seaweed, Nagai Nori USA) held in a small slit cut in a wooden dowel (Fig. 1B). The nori was positioned a fixed distance from the mouth in contact with the lips (Fig. 1C).

For unloaded swallows, seaweed strips with widths one-third the height of the animal's jaw (cut from sheets of sushi nori) were used. The strips were marked at 1 cm intervals using a silver marker pen (Sharpie Metallic Permanent Marker, www.sharpie.com), and externally resembled the inedible seaweed strips shown in Fig. 1D, but without the internal reinforcement. Strips were 14.5 cm long for larger animals and 10.5 cm long for smaller animals.

For loaded swallows designed to measure muscle activation and force, inedible and inelastic seaweed strips were produced (Fig. 1D): sushi nori was applied to both sides of double-sided sticky tape (Scotch Permanent Double-Sided Tape, 3M, Maplewood, MN, USA). Strips were 10.5 cm long and marked with a centimetre scale using a silver marker pen. Strips were trimmed to be one-third of the height of the closed jaws.

### Experimental procedure

Prior to an experiment, animals were gently removed from the main aquarium and weighed. Feeding movements were recorded using a webcam (Logitech HD Pro Webcam C920, Logitech International S.A., Lausanne, Switzerland), swallowing force was recorded on a computer using the data acquisition software AxoGraph (http://axograph.com), and both were analysed using the custom-written software tool Neuroscience Tool for Interactive Characterisation (*neurotic*; Gill et al., 2020), which was used for all measurements based on video and force recordings.

First, animals were induced to generate several bites. The rhinophores (olfactory organs) and anterior tentacles were gently touched with the seaweed stimulus (Fig. 1C). This initiated a strong biting response but did not allow the animal to ingest the seaweed. As the jaws of the animal began to open, the stimulus was removed, so that biting movements could be clearly recorded. To quantify biting behaviour, the overall duration of a bite was measured, subdivided into protraction, measured from the time the jaws opened to peak protraction, and retraction, measured as the time from peak protraction to jaw closure (Fig. 1C).

Second, animals were allowed to swallow seaweed strips. The strips were initially held in forceps until the animal had grasped them, and then the swallowing movements were recorded. Bite-swallows (the initial bite that then became the first swallow) were excluded from the data. The radula typically did not reach the mouth opening and so the duration of the protraction phase could not be accurately measured. Instead, unloaded swallows were measured from the time from which a mark on a seaweed strip began to move towards the jaws until the time that the mark stopped moving inward, i.e. only retraction was measured.

Third, the inedible seaweed was attached to a force transducer (GRASS Instruments Force-Displacement Transducer FT03, Quincy, MA, USA) attached to a magnetic stand, and thus fixed at one end. Forceps were used to guide the free end to the animal's jaws to induce it to swallow. Once the animal had gripped the inedible seaweed, it generated large forces as it attempted to swallow, which consisted of an initial rise, a plateau during which the animal exerted peak forces, and a rapid fall in force as the animal released the inedible seaweed, after which it often made repeated attempts to swallow the inedible seaweed. All data were recorded using AxoGraph (http://axograph.com) and stored in *neurotic* in synchrony with the video data. For small animals, the force transducer was calibrated to 616.7 mV N^−1^, whereas for larger animals, using different springs within the transducer, it was calibrated to 2900 mV N^−1^. To quantify loaded swallows, the following components were annotated: the duration of the force rise and the duration of the force plateau. The entire sequence of bites, unloaded swallows and loaded swallows was repeated three or four times to ensure that many behavioural cycles were obtained.

### Anatomical measurements

After behavioural measures were complete, animals were anaesthetised by injecting isotonic magnesium chloride (333 mmol l^−1^ MgCl_2_) equivalent to half of body mass. The buccal mass was then dissected out and placed in a beaker containing *Aplysia* saline (460 mmol l^−1^ NaCl, 10 mmol l^−1^ KCl, 22 mmol l^−1^ MgCl_2_, 33 mmol l^−1^ MgSO_4_, 10 mmol l^−1^ CaCl_2_, 10 mmol l^−1^ glucose and 10 mmol l^−1^ Mops, pH 7.5). Before being weighed, buccal masses were cut dorsally from the jaw opening through to the oesophagus and dabbed dry with a Kimwipe (Kimtech ScienceBrand, Kimberly-Clark, Roswell, GA, USA).

To produce detailed images of external and internal anatomy, buccal masses ranging in mass from 59 to 2780 mg were imaged using micro-CT. Buccal masses were fixed in an ascending ethanol series (30%, 50% and 70% ethanol in 24 h stages) and stored in 70% ethanol before further processing. Prior to imaging, buccal masses were returned to 30% ethanol (via a 24 h step in 50% ethanol) before being transferred to a contrast agent to enhance X-ray images [0.5% Lugol's iodine (KI/I_2_) in 133 mmol l^−1^ Sorenson's buffer (23.68 g Na_2_HPO_4_ and 18.1 g KH_2_PO_4_ in 1 l deionised water)] to prevent shrinkage (Dawood et al., 2021). Buccal masses were left to steep for 2–4 weeks (depending on size) to achieve even staining throughout the tissue. For scanning, buccal masses were mounted in expanded polystyrene restraints placed inside airtight plastic tubes (85×33 mm), in which pads of water-soaked polyester fibre had been placed above and below the sample to provide a high humidity atmosphere, and imaged using a SkyScan 1172 X-ray micro-CT scanner (Bruker Corporation, Billerica, MA, USA) with a resolution between 2.5 and 5 µm (55 kV source voltage, 180 µA source current, 600–800 ms exposure and 0.1 to 0.2 deg rotation steps). The micro-CT projection images were reconstructed with NRecon (v.1.6.9.18, Bruker Corporation, Billerica, MA, USA). Three-dimensional projections and sections in sagittal and coronal planes were made using Ctvox (v.3.3.0r.1403, Bruker Corporation). Measurements were made of digital sections using ImageJ Fiji (http://imagej.nih.gov/ij).

### Statistical analyses

Averaged measurements are expressed as means±s.d. Scaling relationships were analysed using log_10_-transformed data which converted scaling exponents into a linear form (Clemente and Dick, 2023).

We recorded several bites, swallows or attempts to feed on inedible seaweed from each individual animal (range 2–75 observations per behaviour per animal), producing a range of behavioural durations for each behaviour for each animal (mean number of observations per animal: 19.6 swallows, 32.5 bites and 26.4 attempted swallows of inedible seaweed). To test whether scaling effects of size on behavioural duration were apparent when intra-individual variation in behaviour was accounted for, all data were initially analysed using mixed effect linear models, taking individual behavioural durations per animal as a within-subject repeated measure against mass. As each individual had a unique mass, and this was not a longitudinal study following the same individuals throughout their lifetime, there was no interaction term. Satterthwaite's (1946) method was used to test whether model fits were significantly different from zero (Table 1). These tests were performed using the packages lme4 and lmertest in R (v.4.2.1, http://www.R-project.org/). All models incorporating the individual data were significant (i.e. suggested that the mass of the buccal mass affected behavioural durations, even when considering within-individual variation in timings, and therefore slope≠0). Lines were subsequently fitted on the mean duration values for each behaviour for each animal using reduced major axis (RMA) regression, which is used in scaling studies where values of *y* are not assumed to be causally related to *x*, and where the value of slopes is indicative of scaling relationships (e.g. how length and area relate to volume-correlated mass), as ordinary least squares regression can underestimate slope values (Warton, et al., 2012; Clemente and Dick, 2023). The RMA regressions were performed using the R package SMATR (Warton et al., 2006). This package also tested whether fitted slopes differed from slopes predicted from theory (e.g. differed from the slope predicted from isometric scaling), based on whether residual and axis scores were correlated or not (Warton et al., 2006). Where comparison of model fits was needed, the Akaike information criterion (AIC) was calculated for each model using the aictab() function from the R package AICcmodavg.

**Table 1.**
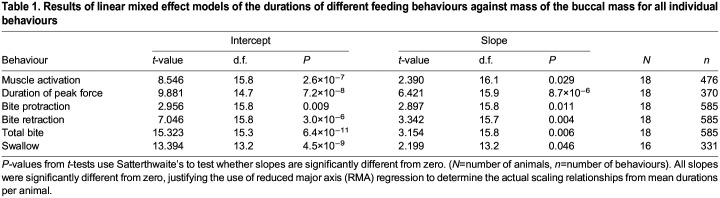
Results of linear mixed effect models of the durations of different feeding behaviours against mass of the buccal mass for all individual behaviours

## RESULTS

### The mass of the buccal mass scales isometrically with body mass

**Fig. 2.**
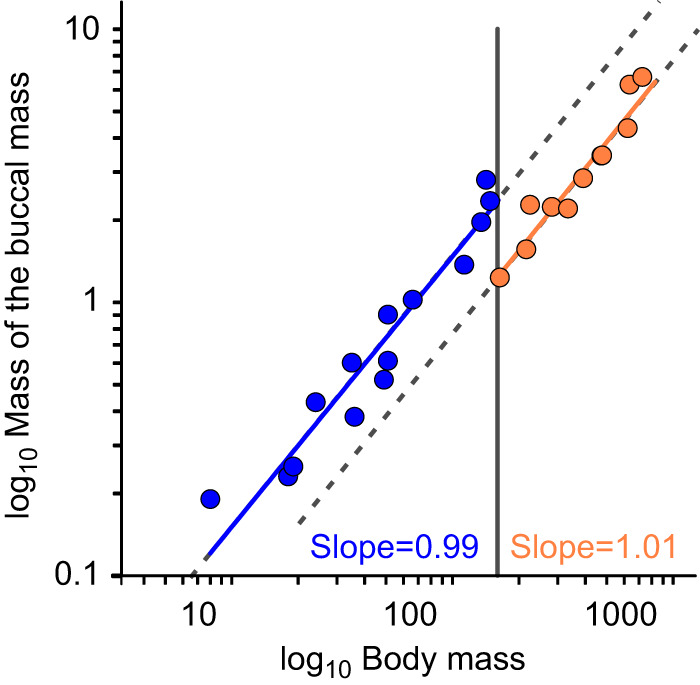
**The buccal mass scales isometrically with body mass prior to and after sexual maturity in *A. californica*.** A log–log plot of the mass (g) of the buccal mass against body mass (g). Sexual maturity occurs when body mass is ∼160 g (dark grey vertical line). The buccal mass scaled isometrically with body mass in both juveniles (blue, *R*^2^=0.93) and adults (orange, *R*^2^=0.95), but there was a change in intercept coinciding with the onset of sexual maturity. The grey dashed lines indicate predicted isometric scaling with a slope of 1.

We measured the mass of the buccal mass from 28 *Aplysia* ranging in body size from 8 to 1424 g (Fig. 2). A slope fitted to all the data using RMA regression had a value of 0.81 (*R*^2^=0.95, *P*=2.2×10^−16^), suggesting a negative allometry. Close inspection, however, suggested that the data formed two linear relationships separated by a disjunction at around 160 g body mass (grey line in Fig. 2). The AIC values of linear models fitted with a single line (AIC=−39.6) as opposed to two lines (AIC=−50.3) suggest that the latter better fits the data. We subsequently found that this disjunction coincides with the onset of sexual maturity (see Discussion). When two lines were fitted, representing immature animals (Fig. 2, blue line, slope=0.99, *R*^2^=0.93, *P*=3.7×10^−8^, slope not significantly different from 1: *r*=−0.04, *P*=0.898) and animals post-sexual maturity (orange line, slope=1.01, *R*^2^=0.95, *P*=5.6×10^−9^, slope not significantly different from 1: *r*=0.06, *P*=0.844), the data suggest that the buccal mass scaled isometrically with body mass, being 1.5±0.3% and 0.8±0.1% of total body mass in juveniles and adults, respectively.

### Feeding in *Aplysia* is quasistatic for all sizes of animal

To determine the relative contributions of kinetic, viscous and elastic forces during feeding in *A. californica*, we estimated the energy allocated to these forces in two hypothetical animals at either end of the experimentally studied body size range: a large (1300 g) and a small (6.6 g) animal, containing isometrically scaled buccal masses that undergo isometrically scaled movements during biting (Table 2). We did not consider gravitational forces, as the buccal mass is fully supported by the mouth, oesophagus and suspensory muscles connecting it to the body wall (Howells, 1942; Chiel et al., 1986).

**Table 2.**
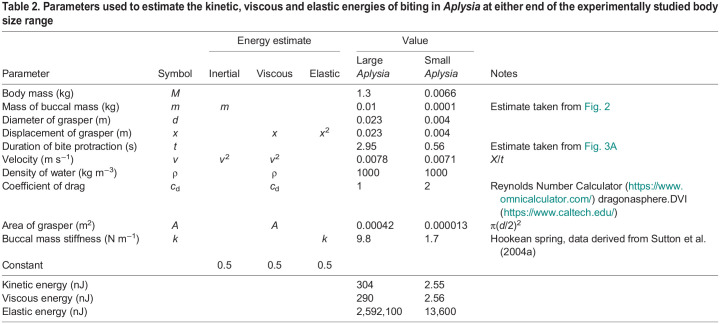
Parameters used to estimate the kinetic, viscous and elastic energies of biting in *Aplysia* at either end of the experimentally studied body size range

Kinetic energy is given by ½*mv*^2^ (Meriam and Kraige, 2012), which we applied using the mass of the buccal mass (*m*) and the displacement (*x*) divided by the bite protraction time (*t*) to estimate velocity (*v*; Table 2). Measuring viscous energy in biological systems can be difficult (Garcia et al., 2000), but this is a standard equation for calculating viscous energy (White, 1994): ½ρ*c*_d_*v*^2^*Ax*. We applied this by calculating the cross-sectional area of the grasper (*A*), using online calculators to estimate the coefficient of drag (*c*_d_; Table 2), and a standard value for the density of water at 10°C (ρ).

The elastic energy (Popov, 1990) is given by ½*kx*^2^, where *k* is the spring constant of *Aplysia*'s musculature. A simplifying assumption is to treat the passive properties of muscles as approximately Hookean springs. Stiffness can then be calculated from force divided by displacement from Hooke's Law. In Sutton et al. (2004a), a grasper from a 250 g animal displaced 0.88 cm, producing a force of approximately 50 mN, yielding a stiffness of 0.05 N/0.0088 m=5.7 N m^−1^. Stiffness scales with length (see below), or mass^1/3^. Therefore a 1300 g animal would be expected to have an estimated stiffness that is 1.73 times greater, 1.73×5.7=9.8 N m^−1^, and a 6.6 g animal would have an estimated stiffness 0.3 times as great, 0.3×5.7=1.7 N m^−1^.

**Fig. 3.**
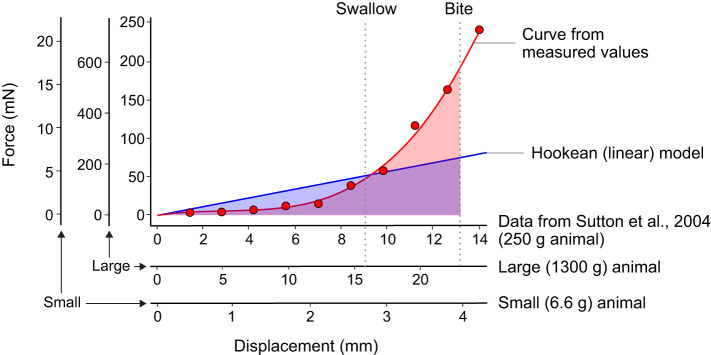
**Force and displacement of the grasper during swallowing and biting in *A. californica.*** The red line shows data measured by Sutton et al. (2004a) for a 250 g animal. The blue line shows an estimate based on a simple linear spring. The elastic energy can be estimated from their respective areas under the graph (red and blue shaded areas). As both stiffness and displacement scale with mass^1/3^, the relationship for both a larger (1300 g) and a smaller animal (6.6 g) is expected to change only in the linear dimensions, as shown by the alternative axes.

A more accurate estimate of the elastic forces during feeding can be extracted from the data in Sutton et al. (2004a), who measured the forces from grasper protraction for a 250 g animal (Sutton et al., 2004a; Fig. 3). The elastic energy is the area under the force/displacement curve (i.e. the integral) from 0 to the protraction distances in swallowing and biting (dotted grey lines in Fig. 3). Compared with the Hookean model (Fig. 3, blue line), the elastic energy of biting is 23.5% greater in the more realistic model (red line), but the energy of swallowing would have been overestimated by a factor of two. The force/displacement graphs for the small (6.6 g) and large (1300 g) *Aplysia* should be isometrically scaled versions of the 250 g animal: hence, the alternative axis scales in Fig. 3 (see below).

From the values calculated in Table 2, only very small amounts of energy are allocated to either inertial or viscous forces (approximately 300 nJ each in the large animal and 3 nJ each in the small), whereas the allocation to elastic energy is four orders of magnitude greater in both the large (2,592,100 nJ) and small (13,600 nJ) *Aplysia*. Using a more complex model of muscle force does not substantially diminish the 10,000-fold gap between elastic energy and kinetic and viscous energies during feeding in *Aplysia.*

In summary, this system is dominated by the interplay between muscle work and elastic energy, and we can neglect inertia (kinetic energy) and damping (viscous energy) in the energy budget throughout the range of body sizes found in *A. californica*.

### Effect of scale on the duration of behaviours governed by quasistatic forces

In quasistatic regimes where elastic forces dominate, the system can be described by *F=kx*, where *F* is force, *k* is the spring constant and *x* is the displacement of a component of the body (Popov, 1990), which during biting in *Aplysia* is the grasper being displaced by about half the length of the buccal mass. The force (*F*) produced by muscle is proportional to its cross-sectional area, i.e. scaling with length^2^ (Clemente and Dick, 2023; Zajac, 1989).

How does the spring constant *k* scale? A muscle can be approximated as a cylinder, where the spring constant is Young's modulus (*E*) times the cross-sectional area of the cylinder divided by its length (Zajac, 1989). If the Young's modulus is scale invariant, then the spring constant will scale proportionately to area/length, or length^2^/length or, simply, length.

If an animal grows isometrically, the displacement (*x*) of body parts during movement will also scale with the animal's characteristic length. Thus, in the equation *F*=*kx*, both muscle force and the product *kx* scale with length^2^, meaning that maximum muscle force increases at the same rate as opposing elastic forces and, if only the biomechanics are considered, large animals will take the same time to generate one cycle of behaviour as small animals. Consequently, any scaling of the duration of behaviours will depend on the scaling of physiological parameters such as muscle activation.

### Muscle activation and force development during swallows on loaded seaweed

**Fig. 4.**
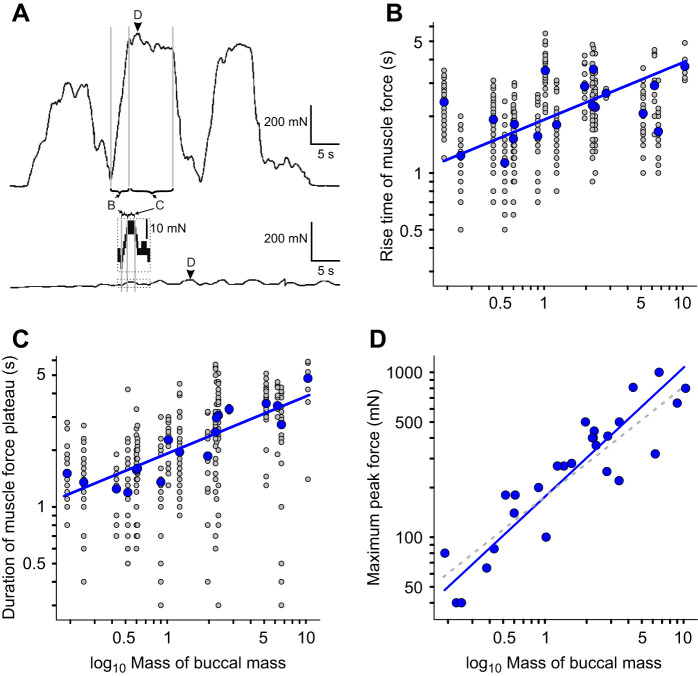
**Timing of muscle activation and force production during attempted swallows of an inedible seaweed strip.** (A) Forces generated by a 1424 g (top) and a 19 g (bottom) animal. Several successive cycles of attempted swallows are shown. The inset shows an expanded trace with the events B–D marked. (B) The time taken for maximum force to be generated at the start of attempted retraction, which was an *in vivo* approximation of muscle activation, plotted against the mass (g) of the buccal mass (*R*^2^=0.27, *P*=0.028). (C) The duration of the plateau of sustained muscle contraction plotted against the mass (g) of the buccal mass (*R*^2^=0.80, *P*=4.6×10^−7^). Blue circles are animal means (to which the lines were fitted; *N=*18) and grey circles indicate individual behaviours. (D) The scaling of maximum force produced by each animal from attempted swallows against mass (g) (*R*^2^=0.81, *P*=1.25×10^−10^). The grey dashed line shows the predicted scaling of force with muscle cross-sectional area (mass^2/3^).

During isometric (length invariant) muscle contractions, where muscle exerts force but does not have a contraction velocity, the timing of contraction depends solely on muscle activation (Zajac, 1989). *Aplysia californica* attempting to swallow tethered and mechanically strengthened inedible seaweed (Fig. 1D) provided a reasonable *in vivo* measure for this isometric condition (see Discussion). The duration of the rising phase of force, from the first muscle contraction up to the maximum force reached at the force plateau (Fig. 4A), therefore revealed the dynamics of muscle activation in the entire buccal mass *in vivo*.

Large forces were generated during attempted swallows. The response consisted of an initial rise in force, as muscle was activated (Fig. 4B), followed by a plateau when maximum muscle force was balanced by the resistance of the inedible seaweed (Fig. 4C). Eventually, the seaweed was released, causing a rapid fall in force before the animal tried again. The rise time of mean force scaled with the mass of the buccal mass^0.3^ (Fig. 4B; mean time to reach maximum force 2.27±0.78 s; RMA regression; *R*^2^=0.27, *P*=0.028, *N*=18). This provided a proxy for how the size of the buccal mass affected muscle activation and generated a quantitative prediction for how the timing of biting and swallowing would scale when the grasper could move freely.

The duration of the plateau of sustained maximum muscle force was similar to that of the rise time (mean 2.35±1.01 s) and scaled with mass^0.37^ (Fig. 4C; RMA regression, *R*^2^=0.80, *P*=4.6×10^−7^).

The peak force that was produced by each animal across all swallows (arrows marked D in Fig. 4A) was taken as an estimate of their maximum muscle force capacity (Fig. 4D). Maximum muscle forces scaled with mass^0.78^ (*R*^2^=0.81, *P*=1.25×10^−10^), which was not significantly different (*r*=−0.35, *P*=0.071) from the scaling of mass^2/3^ predicted by theory that maximum force should scale with muscle cross-sectional area (Zajac, 1989).

### Scaling of bite duration is consistent with activation timing and dominance of quasistatic forces

**Fig. 5.**
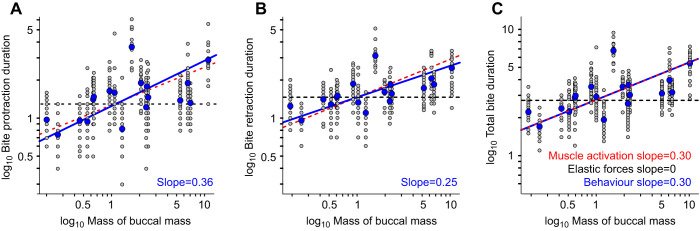
**Bite duration scales with the mass of the buccal mass and follows a time scale similar to that of muscle activation.** (A–C) A log–log plot of the duration (s) of bite protraction (A; *R*^2^=0.34, *P=*0.011), bite retraction (B; *R*^2^=0.41, *P=*0.004) and total bite duration (C; *R*^2^=0.38, *P=*0.006) against the mass (g) of the buccal mass. Grey circles show individual swallows; blue circles show animal means (to which the lines were fitted; *N=*18 in each panel). Slopes were not significantly different from 0.3, the scaling relationship predicted by muscle activation, as shown by the red dashed lines in each panel. The predicted scaling of elastic forces is shown by the black dashed line.

When seaweed strips are not tethered, or *A. californica* has yet to contact food, muscle contractions powering feeding behaviours are not isometric. Instead, muscles will shorten, causing the grasper to move and produce normal feeding behaviours. During bites, protraction was measured from the time the jaws opened to peak grasper protraction, and retraction as the subsequent time from peak protraction until the jaws closed again. Total bite duration was the sum of these two measurements (Fig. 5).

The size of the buccal mass affected the duration of bite protraction (Fig. 5A; RMA regression, *R*^2^=0.34, *P=*0.011, *N=*18). In a buccal mass weighing 0.25 g, protraction took 0.75±0.22 s (*n*=20 bites) whereas in a 10.35 g buccal mass, protraction took 2.89±0.89 s (*n*=23 bites). Therefore, a 41.4-fold increase in mass corresponded to a 3.85-fold increase in protraction duration. Bite protraction duration scaled with mass^0.36^, not significantly different from the mass^0.3^ scaling relationship that we measured for muscle activation (*r*=0.217, *P=*0.386; Fig. 4B).

The duration of bite retraction was likewise affected by the size of the buccal mass (Fig. 5B; RMA regression, *R*^2^=0.41, *P=*0.004, *N=*18). The 0.25 g buccal mass took 0.96±0.15 s to retract, and the 10.35 g buccal mass took 2.48±0.66 s, a 2.6-fold change. Bite retraction duration scaled with mass^0.25^, again not significantly different from mass^0.3^ (*r*=−0.24, *P=*0.340), and it is likely that retraction followed the same scaling relationship as protraction.

Combining protraction and retraction duration to give total bite duration (Fig. 5C) gave a scaling relationship of mass^0.3^, identical to muscle activation (RMA regression, *R*^2^=0.38, *P=*0.006, *N=*18; not different from 0.3, *r*=−0.016, *P=*0.950). Bites lasted 1.7±0.30 s in an individual with a 0.25 g buccal mass and 5.4±1.2 s in a large animal with a 10.35 g buccal mass, a 3.2-fold change.

### Scaling of the duration of unloaded swallows is less than expected from the activation scaling

**Fig. 6.**
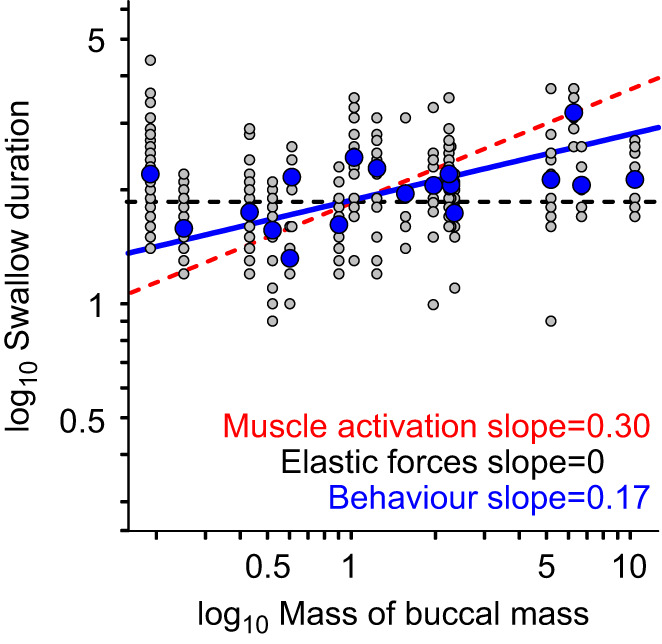
**Swallow duration shows a shallower scaling against size of the buccal mass than predicted from the time constants of muscle activation.** A log–log plot of the duration (s) of inward movement of seaweed during swallows versus mass (g) of the buccal mass, showing a modest effect of size on swallow duration (*R*^2^=0.23, *P=*0.045). Grey circles show individual swallows; blue circles show animal means (to which the line was fitted; *N=*18). The scaling of swallow duration (mass^0.17^, blue line) was significantly less than that predicted from muscle activation (red dashed line). The predicted scaling of elastic forces is shown by the black dashed line.

Swallow duration scaled with mass^0.17^ (Fig. 6; RMA regression, with a low *R*^2^ of 0.23, *P=*0.045, *N=*18), which was significantly lower than the mass^0.3^ scaling of muscle activation (*r*=0.542, *P=*0.020), suggesting that an additional factor was also affecting the scaling relationship. In the 0.25 g buccal mass, swallows took 1.58±0.27 s (*n*=42) and in the 10.35 g buccal mass they took 2.13±0.37 s, (*n*=17), a 35% increase in duration. Swallows and bite retractions were of similar duration in large animals (values at the fitted regression for a 10 g buccal mass were 2.6 s for bites and 2.9 s for swallows, a 12% difference), but the duration of swallows in small animals was much longer compared with bite retractions (for a 0.1 g buccal mass, the equivalent values were 0.8 s for bites and 1.3 s for swallows, a 63% difference). To look for possible explanations, we analysed anatomical scaling relationships (see below).

### Jaw muscles of the buccal mass undergo allometric growth

**Fig. 7.**
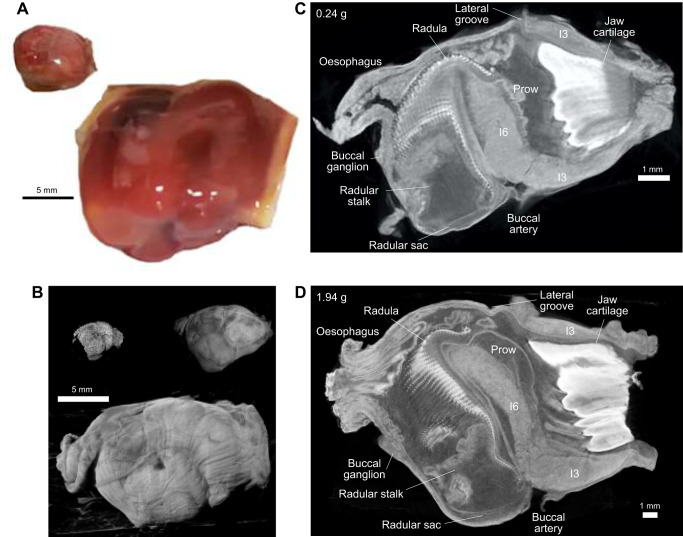
**Anatomical comparison of large and small buccal masses.** (A) A freshly dissected 0.19 g buccal mass from an 8 g animal (left) and a 3.44 g buccal mass from a 477 g animal (right). (B) External views of fixed buccal masses imaged using micro-computed tomography (micro-CT). In ascending order of size, they are 0.06, 0.24 and 2.25 g. (C,D) Mid-sagittal sections of a juvenile (C; 0.24 g) and adult (D; 1.94 g) buccal mass scaled to have the same apparent length. Key anatomical features are labelled.

**Fig. 8.**
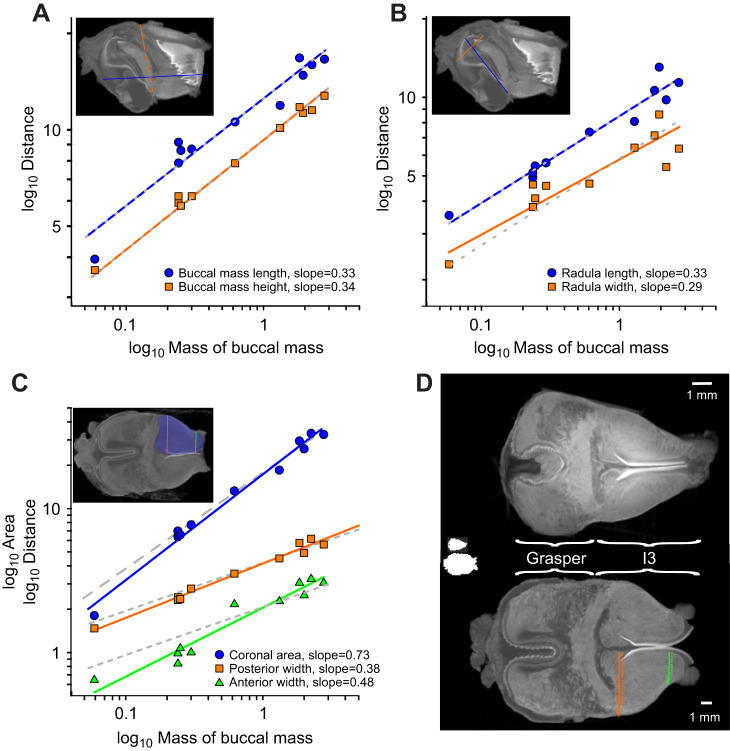
**Isometry and allometry in the anatomy of the buccal mass.** (A–C) Log–log plots of measurements (linear, mm; area, mm^2^) taken from micro-CT scans of 12 buccal masses, as indicated in the inset images, against the mass (g) of the buccal mass. (A) The overall length (blue) and height (orange) scaled isometrically with mass (length: slope=0.334, *R*^2^=0.94, *P=*6.7×10^−7^; height: slope=0.342, *R*^2^=0.974, *P*=1.3×10^−7^). (B) The length (blue) and width of the crown (orange) of the radula also scaled isometrically (radula length: slope=0.333, *R*^2^=0.950, *P*=3.7×10^−7^; radula width: slope=0.288, *R*^2^=0.824, *P*=1.1×10^4^). (C) The large I3 muscle in the anterior buccal mass showed allometric growth. The cross-sectional area of I3 in a mid-coronal view (blue area in inset) scaled with a slope of 0.73 (blue circles; *R*^2^=0.986, *P*=1.4×10^−9^; significantly different from ⅔, *r*=0.63, *P*=0.037). Linear measurements were taken from the rear of I3 near where it joins the grasper (orange squares; slope=0.377; *R*^2^=0.981, *P*=4.4×10^−9^; slope significantly different from ⅓, *r*=0.68, *P*=0.022) and near the anterior margin of I3 (green triangles; slope=0.48; *R*^2^=0.937, *P*=1.0×10^−6^; significantly different from ⅓, *r*=0.88, *P*=1.7×10^−3^), indicating that the allometry was most pronounced in the anterior I3. In each panel the grey dashed lines show the isometric scaling relationship of mass^1/3^ for linear measurements, or mass^2/3^ (long-dashed line in C) for area. (D) Mid-coronal views of a 0.24 g (top) and 2.25 g (bottom) buccal mass scaled to have the same total length, illustrating the difference in the shape of the I3 region in small and large animals. The white insets show the relative sizes of the buccal masses. The orange and green lines indicate the locations of measurements taken for C. The dotted lines indicate the relative length of these measurements in the 0.24 g buccal mass.

The lengths of key structures in the buccal mass (Figs 7C,D and 8) were measured from mid-sagittal and mid-coronal sections of imaged buccal masses ranging from 0.06 to 2.78 g. The overall length of the buccal mass was measured from the site of the attachment of the buccal ganglion to the front of the jaws (blue line in the inset of Fig. 8A) and the total height was measured from the insertion of the buccal artery ventrally to the dorsal indentation of the lateral groove (orange line in the inset of Fig. 8A). Both measurements suggested that overall, the buccal mass grows isometrically, with slopes of near ⅓ in log–log plots against the mass of the buccal mass. For length, the slope was 0.334 (blue Fig. 8A; RMA regression *R*^2^=0.94, *P=*6.7×10^−7^, *N=*11; not different from slope of ⅓, *r*=0.02, *P*=0.961) and for height the slope was 0.342 (*R*^2^=0.974, *P*=1.3×10^−7^; not different from ⅓, *r*=0.18, *P*=0.607).

Similarly, the radula appears to grow isometrically (Fig. 8B). Radula length was measured from its apex to the beginning of the radular sac (blue line in inset of Fig. 8B) and radula maximum width was also measured (orange line in inset of Fig. 8B). Radula length scaled with mass^0.333^ (*R*^2^=0.950, *P*=3.7×10^−7^; slope not different from ⅓, *r*=−0.002, *P*=0.995), and radula width scaled with mass^0.288^ (*R*^2^=0.824, *P*=1.1×10^−4^; not different from a slope of ⅓, *r*=−0.33, *P*=0.321), both consistent with isometric growth.

In contrast, the anterior compartment of the buccal mass, which is predominantly formed from the large I3 muscle (Howells, 1942), showed pronounced allometric growth. The I3 muscle runs from the anterior edges of the grasper to the front extremity of the jaw (Figs 7C,D and 8C,D). Although the anterior–posterior length of I3 scaled isometrically with the mass of the buccal mass (data not shown; slope=0.322, *R*^2^=0.827, *P*=1.0×10^−4^; not significantly different from a slope of ⅓, *r*=−0.08, *P*=0.820), there were clear differences in its anterior proportions. In small *A. californica*, the buccal masses were pear shaped, with the width of the I3 tapering anteriorly, whereas in mature animals the buccal masses were more barrel shaped with less anterior reduction in width (compare external, sagittal and coronal views in Figs 7 and 8D). The cross-sectional area of I3 in a mid-coronal view (Fig. 8C, blue area in inset) scaled as mass^0.73^ (Fig. 7C; blue circles; *R*^2^=0.986, *P*=1.4×10^−9^), significantly different from the isometric expectation that area should scale against volume^2/3^ (*r*=0.63, *P*=0.037). The posterior width of I3, near where it joins the grasper (Fig. 8C, orange squares) had a slope of 0.377 (*R*^2^=0.981, *P*=4.4×10^−9^), which was only slightly but still significantly different from the isometric expectation of ⅓ (*r*=0.68, *P*=0.022). The anterior width of I3, however (Fig. 8C, green triangles), had a steeper scaling relationship with a slope of 0.48 (*R*^2^=0.937, *P*=1.0×10^−6^; significantly different from isometry, *r*=0.88, *P*=1.7×10^−3^), which suggests that the allometry is most pronounced in the anterior I3, in accordance with the observed differences in the shapes of large and small buccal masses. This allometric growth of I3 could account for the scaling of swallows (see Discussion).

## DISCUSSION

### Energy budgets predict that feeding behaviour in *Aplysia* is dominated by quasistatic forces

The overall scaling of a behaviour depends on the scaling of both mechanical forces (Sutton et al., 2023) and physiological processes, which we analysed by comparing feeding behaviour in *Aplysia* across its lifespan. The MPS framework (Sutton et al., 2023) predicts that feeding by all sizes of *Aplysia* should be dominated by elastic (quasistatic) forces, which was supported by our energy budget calculations. The 10,000-fold magnitude by which elastic energy exceeded kinetic and viscous energies in both small and large animals would be largely unaffected even if kinetic and viscous energies were doubled to cover both protraction and retraction, or if it were recognised that retraction may be assisted by the passive return of elastic energy (Sutton et al., 2004a), or if the stiffness of muscles had been overestimated by using an approximation to a Hookean spring.

Because elastic forces dominate, the scaling of the elastic forces resisting movement (scaling with mass^2/3^; Zajac, 1989) equals that of the muscular forces producing movement (scaling with mass^2/3^), cancelling each other out. Based on purely mechanical considerations, therefore, there should be no scaling effect on feeding duration during growth. This is mathematically identical to the static similarity model of McMahon (1975).

If instead we hypothesise that inertial forces were the most important component in the behaviour, then, because the scaling of muscular forces (mass^2/3^) is less than that of mass itself (mass^1^), there will be a slowing of behaviour by a factor of mass^1/3^ (akin to the geometric similarity argument in McMahon, 1975, and suggested by Hill, 1950). Assuming a similar scaling of muscle activation (mass^0.3^), this would give a final scaling of duration of mass^0.63^, much greater than what we measured.

It is more difficult to predict the scaling exponent for behaviours dominated by internal viscous forces because few studies have attempted to quantify them. Garcia et al. (2000) proposed that viscous damping forces in tissues should scale with mass^1/3^. Accordingly, muscle force will increase more rapidly than viscous forces with scale, causing behaviours to occur more rapidly. Combining viscous force scaling with the effect of slowing muscle activation with scale (proportional to mass^0.3^) gives a final scaling factor that is near-zero, proportional to mass^−0.03^, again different from our measured scaling relationship. These counterfactual cases show how the scaling of both mechanical forces and physiological processes determines how growth affects behaviour.

### The timing of feeding behaviour in *Aplysia* is dominated by the dynamics of muscle activation

In the absence of any mechanical scaling effects, the scaling of the duration of *Aplysia* feeding behaviour should predominately reflect the scaling of muscle activation. We found that the time to reach maximum muscle activation during feeding in *A. californica* scaled with mass^0.30^ by using attempts to feed on inedible seaweed as an *in vivo* approximation of isometric muscle activation in the buccal mass (Fig. 3B). We then compared this with the scaling of normal feeding behaviours, where non-isometric muscle contraction produced movements in the buccal mass. The scaling of bite duration was the same as that of muscle activation (Fig. 4), further supporting the hypothesis that elastic forces dominate in *Aplysia* feeding. In contrast, swallowing showed shallower scaling, proportional to mass^0.17^ (Fig. 5). We suggest that allometric growth of the anterior I3 muscle, responsible for grasper retraction (Lu et al., 2015; McManus et al., 2014), allows greater forces to be exerted at a lower muscle activation in larger animals (Fig. 7; see below).

### Why does muscle activation show scale-dependent properties in *Aplysia*?

*Aplysia* muscles, like those of most invertebrates, have few motor neurons (<10) which branch extensively across the muscle, becoming finer and slower as they do so (Katz, 1949; Carew et al., 1974; Burrows, 1996). Furthermore, most muscles in *Aplysia* are not electrically excitable and depend on local motor neuronal excitation to activate contraction (Cohen et al., 1978), in contrast to skeletal muscles in vertebrates and arthropods (Pichon and Ashcroft, 1985; Catterall, 1991). It is therefore likely that neuronal branch length strongly affects muscle activation, which may proceed sequentially, spreading across the muscle. Molluscan motor neuron arborisations and the distribution of motor end plates will be broadly proportional to the surface area of the innervated muscle (scaling with mass^2/3^), whereas the muscle itself scales with volume (scaling with mass^1^), creating a mismatch between presynaptic excitation and the passive spread of depolarisation within the muscle, suggesting a possible explanation for the scaling of muscle activation as close to mass^1/3^. Axon lengths should scale with body length, also potentially contributing to a mass^1/3^ scaling in activation timing. Furthermore, in unmyelinated invertebrate neurons, conduction velocity is proportional to axon diameter^1/2^ (Hartline and Colman, 2007), meaning that increased conduction speeds to compensate for increased axon lengths can only be achieved through allometric growth of axon diameters, perhaps explaining the very large somata size of many *Aplysia* motor neurons (Kandel, 1979).

### Anatomical scaling relationships

The buccal mass scaled isometrically with body mass, except for a small disjunction centred at ∼160 g body mass, near the size at which *A. californica* attains sexual maturity (Gerdes and Fieber, 2006). After sexual maturity, body mass will be invested in gonads and eggs (the animals are hermaphrodites). Consequently, the buccal mass becomes a smaller proportion of body mass, and this is reflected in a downward shift of the line intercept, without affecting its slope. As sexual maturation is a comparatively rapid process, it was unlikely that maturing animals between the two growth trends would be captured, giving the appearance of two distinct populations. Possibly, the transition can be triggered in body sizes ranging from ∼120 to 180 g, hence the apparent ‘drop’ of the intercept (Fig. 2).

An important exception to the overall isometric growth of the buccal mass was the disproportionately larger size of the I3 muscle in larger animals. Swallowing is powered by strong contractions of I3 (McManus et al., 2014, 2019; Lu et al., 2015), which may need to activate more fully in juveniles before it can produce a force equivalent to that of a large animal. As activation is time dependent, small animals will take longer to achieve sufficient force to allow swallowing. This may explain why the scaling of swallow durations was lower than that predicted from muscle activation. The deviation of the scaling of the anterior I3 from isometry (mass^0.15^) is similar to the deviation of the scaling of swallowing from that of muscle activation (mass^0.13^). In our experiment, seaweed was untethered, but in nature, seaweed is generally attached to a substrate. Small animals are restricted to eating small seaweeds that are likely less tough and more loosely anchored. Larger animals eat a larger variety of seaweeds than juveniles (Pennings, 1990), which may be tougher, more firmly attached and more difficult to manipulate as they occur in different forms [e.g. sheets in sea lettuce (*Ulva*), spherical in sea grapes (*Halosaccion*), and long cylindrical stipes in the red alga *Gracilaria*]. Moreover, adults may have to deal with seaweed anti-herbivory strategies, both morphological and chemical (e.g. calcification; Duffy and Hay, 1990). The retractor muscle hypertrophy may be needed to deal with these more difficult foods. In another molluscan species, the cuttlefish (*Sepia officinalis*), buccal mass growth is apparently isometric despite diet changes between juveniles and adults (Souquet et al., 2023).

In contrast to swallowing, protraction during biting is largely produced by the I2 muscle at the rear of the buccal mass (Hurwitz et al., 1996; Yu et al., 1999). As the rear of the buccal mass showed isometric anatomical scaling, the scaling of protraction duration during biting was similar to that of muscle activation.

### Sources of variability in feeding behaviour

Our *A. californica* were highly motivated to feed, having been previously food deprived, but all feeding behaviours showed relatively large intra-individual variances (grey circles in Figs 3–5). Such variability is characteristic of *Aplysia* feeding both behaviourally and in the underlying neural patterning (Cullins et al., 2015a). Sensory feedback also contributes to this variability as it updates motor patterns in response to previous movements (Cullins et al., 2015b). Grasper movement is under less constraint than legged locomotion, where stability must be maintained: an animal will fall if an incorrectly positioned leg cannot bear weight in stance. *Aplysia* feeding behaviour has no such stability constraints. There is little mechanical constraint determining the movement range in each cycle beyond the maxima that the grasper can be protracted or retracted (Neustadter et al., 2002; Drushel et al., 1997; 1998). Thus, rapid behavioural times may not represent the fastest behaviour, but rather cycles with smaller grasper excursions, justifying our use of mean durations.

Most grasper movement was unseen during feeding. During biting, observations were limited to when the jaws were open. During swallowing, inward movement of the seaweed was used to measure grasper retraction. These limitations may have contributed to data variability.

The rising phase of force during the attempted swallows of tethered seaweed was not a perfect measure of total muscle activation. During the first few cycles there was some inward movement of the seaweed but, once taut, further movement of seaweed or muscle was impossible, and repeated cycles of muscle contraction occurred with minimal muscle velocity (Gill and Chiel, 2020). Nevertheless, during each attempt, the grasper released the seaweed strip (Gill and Chiel, 2020), potentially allowing small movements, which violates the requirement for strictly isometric contractions to accurately measure muscle activation.

Extrinsic buccal mass muscles position and anchor the buccal mass against the lips and greatly assist feeding by creating a solid block of tissue (Chiel et al., 1986). In addition, the head can move relative to the foot, and the whole animal can move backwards to exert force on food items (Kupfermann, 1974). Our *in vivo* measurements of muscle activation and behaviour reflect the activity of many muscles, within and outside the buccal mass, which have considerable freedom in their extent of involvement. Analysing the buccal mass in isolation severs these essential extrinsic muscles and negatively affects feeding behaviour (Chiel et al., 1986), while our whole-animal measurements are relevant for natural behaviour.

### The control of behaviour under different force regimes

Behaviour cannot be fully understood without accounting for the action of the nervous system, muscle properties and the biomechanics of the body in which this occurs (Chiel and Beer, 1997). Body size is fundamental to understanding the dominant forces acting during behaviour, and the complexity of the tasks that the nervous system must perform to control movement and maintain stability.

In large animals, where inertial and gravitational effects are substantial, legged locomotion can be passively assisted by gravity and momentum but is inherently unstable, and recovery from unexpected disruptions can require multi-step adjustments to prevent falling (Hooper, 2012). This complexity, together with longer neuronal conduction times, requires the central nervous system to anticipate muscle activation, rather than simply react to sequences of internal and external stimuli (More et al., 2010). Furthermore, as length and mass affect the momentum and pendulum-like oscillations of legs, neural circuits controlling movement in growing animals must be constantly modified, potentially even having to adjust to an altered preponderance of inertial and elastic forces.

In contrast, controlling behaviours governed by quasistatic forces may be simpler. First, quasistatic systems are inherently stable: cessation of motor neuron activity instantly stops ongoing motion and either the system arrests or elastic forces return the system towards a rest position, which tend to be structured to facilitate ongoing movement cycles (Guschlbauer et al., 2022; Kubow and Full, 1999). Second, relative forces in large and small animals are biomechanically identical and time invariant so little alteration in neuronal circuitry may be needed during growth in the absence of muscle activation scaling effects. For example, an identical number of neurons and no size-dependent variation in cycle period and the relative phasing of neural activity were found in the neuronal circuitry controlling the stomachs of adult and juvenile lobsters (Bucher et al., 2005). In *Aplysia*, however, numbers of central neurons are known to increase during growth (Cash and Carew, 1989).

The increased stability afforded by quasistatic systems comes at the cost that all active movement must be powered by ongoing muscle contraction, which ultimately determines the timing of behaviour. As shown by the buccal mass of *Aplysia*, the slow activation of muscles is the principal determinant of the duration of behavioural cycles during growth and does much to shape the final behavioural pattern.

In conclusion, understanding how behavioural cycles scale with size requires analysis of both the underlying mechanical forces, for which the MPS framework (Sutton et al., 2023) is a valuable conceptual tool, and an analysis of physiological processes such as muscle activation. For small-magnitude, slow-moving behaviours, where elastic forces dominate, and viscous and inertial forces can be neglected, the mechanics of movement will not affect the frequency of behaviour of animals of different sizes. In contrast, behavioural frequency will be strongly affected by size-dependent changes in muscle activation. We have demonstrated that this relationship can hold across three orders of magnitude of size. Allometric growth of body structures is one way in which the constraints imposed by the muscle activation and force regime scaling can be altered, thus changing the duration of behavioural cycles. Provided that the animal does not become large enough or move fast enough that inertia or viscosity strongly affects the mechanics of movement, muscle activation will be the major determinant of the frequency of behaviour.
